# Optimisation of railway tamping scheduling

**DOI:** 10.1016/j.heliyon.2024.e40844

**Published:** 2024-11-30

**Authors:** Mahdi Khosravi, Alireza Ahmadi, Ahmad Kasraei, Arne Nissen

**Affiliations:** a Department of Civil, Environmental and Natural Resources Engineering, Luleå University of Technology, Luleå, Sweden; b School of Aviation, Australian University, West Mishref, Kuwait; c Trafikverket, Luleå, Sweden

**Keywords:** Railway tracks, Track geometry degradation, Maintenance, Optimal tamping scheduling, Track geometry modelling, Track geometry measurement alignment

## Abstract

This research was devoted to optimising opportunistic tamping scheduling to present a cost-effective approach that considers both preventive and corrective tamping activities. To achieve this, we formulated the track geometry tamping scheduling problem as a mixed integer linear programming model and employed a genetic algorithm for its resolution. Key track quality indicators, including the standard deviation of the longitudinal level and single defects, were considered.

We developed predictive models for the evolution of standard deviation and single defects over time, which were utilised to schedule preventive tamping activities and anticipate potential corrective actions. Additionally, we investigated the impact of both preventive and corrective tamping activities on the values of standard deviation and single defects.

A case study on data from the Main Western Line in Sweden demonstrated that the fixed cost of occupying each maintenance window significantly influenced the total tamping cost. Moreover, the maintenance cycle interval notably affected the number of required corrective tamping activities. Specifically, a 3-month interval led to over 50 % fewer corrective tamping activities when compared to a 9-month interval. The results revealed that a 6-month interval achieved a favourable balance between corrective and preventive tamping activities and the total cost in our case study.

## Highlights

• Optimal tamping scheduling minimises occurrences of multiple single defects.
• Optimal scheduling reduces maintenance windows, grouping tamping activities.
• Total tamping cost highly impacted by maintenance windows fixed costs.
• Sensitivity analyses show importance of maintenance intervals on costs.
• Incorporating unused segment life costs is key for efficient tamping.

**Table:** Abbreviations and nomenclature used in this paper

Abbreviation	Description	Abbreviation	Description
CM	Corrective maintenance	OM	Opportunistic maintenance
GA	Genetic algorithm	PM	Preventive maintenance
IAL	Immediate action limit for standard deviation	UH1	Lower bound for CM
MFCOW	Modified correlation optimised warping with channel fusion	UH2	Upper bound for CM
MILP	Mixed integer linear programming	KS	Kolmogorov–Smirnov

Notations	Description	Notations	Description

$C^{PM}$	Cost of PM	$a_{0}$, $\alpha_{0}$	Regression coefficients
$C^{ϻ}$	Cost of the unused life of segments tamped early	$a_{1}$, $a_{2}$, $a_{3}$, $\alpha_{1}$, $\alpha_{2}$	Coefficients for the explanatory variables in linear models
$C^{UH1}$	Cost of CM	$b_{s}$	Degradation rate of SL in segment s
D	Value of a single defect	$\beta_{s}$	Degradation rate of D in segment s
$D^{i}$	$i^{th}$ peak with the absolute highest value in a segment	γ	UH1 or lower bound for CM
${D_{s,t}}^{i}$	$i^{th}$ peak with the absolute highest value in segment s at time t	δ	Alert limit for standard deviation
${D_{s,0}}^{i}$	${D_{s,t}}^{i}$ at time ͳ	η	Planning limit for single defects
${D_{s,t}}^{i+}$	Value of ${D_{s,t}}^{i}$ immediately after tamping	Θ	Vector of occupied maintenance windows
${D_{s,t}}^{i-}$	Value of ${D_{s,t}}^{i}$ immediately before tamping	$\Theta_{w}$	Binary variable, which is 1 if the maintenance window w is occupied with at least one tamping activity, and 0 otherwise
M	Number of months for the scheduling finite time horizon	$\Theta^{*}$	Optimal maintenance window vector
${R_{s,t}}^{SL}$	Recovery value of ${SL}_{s,t}$	κ	Tamping matrix
${R_{s,t}}^{D^{i}}$	Recovery value of ${D_{s,t}}^{i}$	$\kappa_{s,w}$	Binary variable, which is 1 if the track segment s is tamped in maintenance window w, and 0 otherwise.
S	Number of segments	$\kappa^{*}$	Optimal tamping matrix
SL	Standard deviation of the longitudinal level	$\vartheta_{s}$	Binary variable, which is 1 if track section s is on a curve, and 0 otherwise
${SL}_{s,0}$	${SL}_{s,t}$ at time ͳ	ξ	Intervention limit for standard deviation
${SL}_{s,t}$	Standard deviation in segment s in time t	ρ	Fixed possession time for each tamping window
${{SL}_{s,t}}^{-}$	Value of ${SL}_{s,t}$ immediately before tamping	$\phi_{s,w}$	Matrix for the number of cooling-down and warming-up events
${{SL}_{s,t}}^{+}$	Value of ${SL}_{s,t}$ immediately after tamping	ψ	A dummy variable that denotes types of tamping
$T_{w}^{PM}$	Total tamping time	$L_{s}$	Length of segment s
$T_{w}^{\Omega }$	Time required for tamping a segment	ȴ	Interval of each maintenance cycle
$T_{w}^{\upsilon }$	Travelling time required to reach the segments that need tamping	f	Fixed cost of occupying each tamping window
$T_{w}^{\omega }$	Time spent for warming-up and cooling-down for window w	ʆ	Number of peaks to be considered as quality indicators in each segment for the scheduling problem
$T_{\omega }$	Time spent for warming-up and cooling-down	ͳ	Time of the latest tamping intervention
$V_{\Omega }$	Speed of the machine whilst tamping	${Ц}_{s,t_{w}}$	Unused life of the segments in each tamping window
$V_{\upsilon }$	Speed of the tamping machine whilst travelling to reach the segments that need tamping	℘	Corrective tamping matrix
W	Number of tamping windows in the scheduling finite time horizon	${℘}_{i,s,m}$	Binary variable, which is 1 if the $i^{th}$ defect value of longitudinal level for segment s at time $t_{m}$ is tamped, and 0 otherwise.

## 1 Introduction

Railway track geometry deteriorates over time due to a variety of factors, including traffic loading and environmental conditions. Track degradation has negative consequences for safety, track availability and passenger comfort [1]. To control degradation and restore railway track geometry conditions to an acceptable level, maintenance is required. Tamping is one of the primary maintenance activities undertaken to recover track geometry conditions. An appropriate tamping scheduling regime is crucial to maintain track geometry quality at an acceptable level [2]. Additionally, it positively affects the track availability and capacity. Tamping scheduling deals with arranging, controlling and optimising work and workloads.

Two types of tamping are generally performed on track segments: corrective maintenance (CM) and preventive maintenance (PM). CM activities are typically performed by small tamping machines and only cover a small portion of the segment, whilst PM activities generally cover the entire segment. CM activities recover the track to an operational state after the occurrence of single defects, which are short irregularities in track geometry that may accelerate the occurrence of rail defects [3]. These single defects are identified as peaks with values higher than a predefined threshold in the longitudinal level data points. PM activities are scheduled tamping activities that are performed to avoid unexpected defects on the track. Unlike CM, PM activities are scheduled to be performed within predefined maintenance windows and do not disrupt service operations. A proper scheduling strategy should avoid the occurrence of CM between maintenance windows, which significantly increases the safety and availability of the track at a lower cost.

Opportunistic maintenance (OM) is a form of maintenance scheduling that can significantly improve the performance and cost-effectiveness of scheduling and planning whilst improving track availability. OM takes advantage of planned maintenance windows during which suitable resources are already available. OM provides the opportunity to tamp track segments that are found to pass the degradation limits in the near future or are susceptible to the occurrence of single defects. The OM strategy groups segments together for tamping actions based on three types of dependence: structural, stochastic and economic. Considering these dependence types, tamping may be performed on more segments than necessary in a specified time to reduce future tamping needs. Considering the economic dependence, it may be cost-effective to perform the tamping of some segments earlier or postpone it [[4], [5], [6]]. This can reduce the cost of logistics and available machines used for tamping. Considering the stochastic dependence, the degradation of one segment may affect the degradation of adjacent segments [[7], [8], [9]]. Additionally, considering the structural dependence, the tamping of one segment may affect the status of neighbouring segments due to the physical interactions among them [1,2]. Gustavsson [6] considered OM from the perspective of economic dependence by incorporating setup costs in the model. Letot et al. [10] applied OM by grouping track sections with intervention times close to the tamping time. Pargar et al. [11] applied a technique for grouping and balancing maintenance actions to minimise maintenance and renewal costs. Additionally, Khajehei et al. [2] integrated OM in their proposed scheduling model with consideration for economic and structural dependence. This was achieved by establishing a fixed cost associated with the occupation of tamping windows and introducing a constraint mandating tamping on a track section positioned between two sections requiring maintenance. In general, incorporating OM strategies can lead to a more efficient scheduling strategy.

Numerous research studies have been devoted to optimising the scheduling of tamping activities. Based on the available literature, using the standard deviation of longitudinal level (SL) is the dominant track quality indicator among studies involving the scheduling of tamping activities [2,12,13]. However, a few studies have also considered the standard deviation of horizontal level or other track geometry parameters as quality indicators [14,15].

Furthermore, in the realm of modelling track geometry degradation, two distinct approaches are commonly employed: deterministic and stochastic. The deterministic models documented in the literature are mostly linear [6,13,16] and exponential [2,17]. Some studies have modelled the degradation of track geometry as a stochastic process using the gamma process [18,19], Markov chain [14], Wiener process [20] or a combination of using Monte Carlo simulation and distribution functions [13,21].

Additionally, researchers have considered different objectives for scheduling maintenance activities. Some researchers considered different types of maintenance activities (e.g. grinding and tamping) in their scheduling problems and attempted to minimise the total cost of maintenance. Some of these studies attempted to simultaneously minimise the track possession [[22], [23], [24]] or renewal cost [11] while minimising the total cost of maintenance activities. Many studies have considered the total cost of tamping as the objective function for scheduling tamping activities [16,25,26]. Additionally, Vale, Ribeiro et al. [27] considered minimising the total number of tamping activities as the objective function. Some studies have considered multi-objective functions. Lee, Choi et al. [12] proposed two objective functions to minimise both tamping cost and the number of tamping interventions. Bressi, Santos et al. [15] attempted to minimise the life cycle maintenance costs and maximise the life cycle quality level of the track considering different levels of reliability. Chang, Liu et al. [28] considered total maintenance cost, window levelling and resource levelling as optimisation objectives. Additionally, Kasraei and Zakeri [13] attempted to maximise track availability whilst minimising the total tamping cost. Some studies have also attempted to take advantage of predicting the probability of a single defect occurrence. He, Li et al. [29] proposed two objective functions for minimising the risk of derailment and cost of single defect rectification via the prediction of the degradation process of single defects. Sharma, Cui et al. [14] considered the probability of geometry defect occurrence using a track quality index of multiple track geometry parameters and attempted to minimise the cost of CM. Furthermore, Khajehei, Haddadzade et al. [2] considered the probability of single defects occurring by using the standard deviation of the longitudinal level and attempted to minimise the total cost of tamping whilst having the lowest number of CM activities.

Moreover, various approaches have been employed to solve the problem of optimisation. Meier-Hirmer, Carolina, Riboulet et al. and Sharma, Cui et al., 2018 [14,19] developed a Markov decision process for tamping decision making. Additionally, Kasraei and Zakeri [13] applied a Monte Carlo technique to estimate the number of maintenance operations. Many existing studies have formulated the problem of scheduling tamping activities by mixed integer linear programming (MILP) and solved such problems using metaheuristics algorithms such as a genetic algorithm (GA) [2,15,17] or simplex algorithms such as Cplex [11,25,30]. Generally, for problems with large sizes, metaheuristics methods are suggested because computational time increases exponentially when the number of components and the number of time periods increase.

This study aimed to present cost-effective tamping scheduling by considering the effects of both PM and unavoidable CM activities on the tamping strategy. Considering the aforementioned literature, in this study we tackle following gaps.

• A few studies have considered the probability of single defect occurrence in tamping strategies. However, one of the novelties of this paper is predicting the value of single defects (D) over time for detecting possible CM activities. Therefore, the values of standard deviations and single defects for each segment were considered as track quality indicators. Linear functions were then used to model the evolution of standard deviation and single defects over time. The predicted values of these indicators were being used as a prerequisite for scheduling PM and predicting possible required CM activities.
• Additionally, when investigating tamping strategies, it is important to also consider the effects of recovery by both PM and possible CM activities on the values of standard deviation and single defects in the track segments. Accordingly, in this study, tamping recovery for both PM and CM activities were modelled to estimate the values of standard deviation and single defects after tamping.
• Furthermore, since the geometry measurements suffer from an uncontrolled shift due to stretching or compression of the measurements, this shift can lead to the incorrect positioning of single defects and falsifies the analysis of their evolution patterns [[31], [32], [33]]. Therefore, to increase the accuracy of single defect values, track geometry measurements must be aligned at a high resolution. For this purpose, an alignment method known as modified correlation optimised warping with channel fusion (MFCOW) was first employed to correct variations in the positions of single defects.
• Finally, an optimisation approach was developed to minimise the total cost of tamping using an OM strategy within a specified time horizon.

The scheduling problem was formulated as a MILP model and a GA was implemented to achieve optimal tamping scheduling. GA is one of the most popular metaheuristics algorithms and has wide applications for solving scheduling problems [2,34]. To evaluate the proposed approach, a case study was conducted on the data collected from a line section of the Main Line in Sweden.

The remainder of this paper is organised as follows. In Section 2, the tamping scheduling problem is described in detail. The methodology proposed to deal with the problem is described in Section 3. Then, Section 4 presents a case study and Section 5 illustrates the results of the application of the proposed methodology on the case study. Finally, conclusions related to the outcomes of the research and suggestions for future research are provided in Section 6.

## 2 Problem description

This study investigated a cost-effective tamping schedule for a track line with S number of segments with different lengths ($L_{s}$ sϵS) in a finite time horizon (i.e. M months). In the given time horizon, each segment can be considered for PM tamping in W number of tamping windows with fixed possession times (ρ). W is calculated using the following equation:

(1) W=M/ȴ

Where ȴ is the determined number of months between two tamping windows or the interval of each maintenance cycle. Based on W and S, and whilst considering the segments that require tamping in each tamping window, the tamping matrix κ can be formed as follows:

(2) $$\kappa =\left(\begin{array}{ccc} \begin{array}{cc} \begin{array}{c} \kappa_{1,1}\\ \kappa_{2,1} \end{array} & \begin{array}{c} \kappa_{1,2}\\ \kappa_{2,2} \end{array} \end{array} & \cdots & \begin{array}{c} \kappa_{1,W}\\ \kappa_{2,W} \end{array}\\ \vdots & \ddots & \vdots \\ \begin{array}{cc} \kappa_{S,1} & \kappa_{S,2} \end{array} & \cdots & \kappa_{S,W} \end{array}\right)$$

where cells in this matrix $\kappa_{s,w}$ (sϵS and wϵW) are binary decision variables and have been defined as 1 if the track segment s is tamped in maintenance window w, and 0 otherwise. In each tamping window, if at least one segment is considered for tamping, a fixed cost (f>0)—including the cost of equipment and crew available for maintenance—should be borne. Therefore, it may be more cost-effective to not occupy some maintenance windows for tamping activities. Accordingly, vector Θ is formed to show the occupied maintenance windows:

(3) $$\Theta =\left(\begin{array}{c} \Theta_{1}\\ \begin{array}{c} \Theta_{2}\\ \vdots \end{array}\\ \Theta_{W} \end{array}\right)$$

where $\Theta_{w}$ refers to each row of this vector and is a binary decision variable that gets 1 if the maintenance window w is occupied with at least one tamping activity, and 0 otherwise. The number of occupied maintenance windows can affect the maintenance cost. This creates an opportunity to postpone or push forward the tamping of a segment to group tamping activities in less tamping windows and reduce the number of occupied tamping windows. This OM strategy significantly reduces maintenance costs whilst potentially increasing track capacity. However, postponing the tamping of a segment may lead to a CM, whilst pushing it forward results in increasing the unused life of the segment due to early tamping. CM is more expensive than PM and increasing the unused life is costly because it reduces the life cycle of the segment [2]. Therefore, it is important to consider a trade-off between the cost of unused segment life, the cost of potential CM and the fixed cost of occupying a tamping window when planning tamping activities.

For planning tamping activities, in this study, the standard deviation values and single defects, were considered as indicators. It should be noted that to assess the single defects values, in this study, we track the peaks with the highest values in the longitudinal level data points for each segment. The number of peaks to be considered is ʆ $\left(D^{i},i=\left\{1,2,\ldots ,ʆ\right\}\right)$, where ʆ/2 of them are peaks with the highest maximums and ʆ/2 of them are peaks with the lowest minimums. To plan and conduct maintenance actions, Trafikverket (the Swedish Transport Administration) has defined three limits for the value of standard deviation and four limits for the value of single defects [35]. Accordingly, the limits for standard deviation are as follows: the alert limit (δ); intervention limit (ξ); immediate action limit (IAL). Meanwhile, the limits for single defects are as follows: planning limit (η); lower bound for CM or UH1 (γ); upper bound for CM (UH2); critical limit. According to these limits, the rules listed in Table 1 were applied when considering a track segment as a candidate for upcoming tamping activity in this study.

**Table 1:** Decisions related to considering a track section for tamping.

Condition	Decision
$\left({SL}_{s,t}>\xi \;|\;\max\left({D_{s,t}}^{i},i\in ʆ\right)>\gamma \right)$	The track segment s must be tamped without any delay
$\left({SL}_{s,t}>\delta \;|\;\max\left({D_{s,t}}^{i},i\in ʆ\right)>\eta \right)$&$\left({SL}_{s,t}<\xi \;\&\;\max\left({D_{s,t}}^{i},i\in ʆ\right)<\gamma \right)$	The track segment s can be considered for tamping based on the OM strategy

Considering Table 1, when the value of SL or one of $D^{i},i\in ʆ$ passes intervention limit and UH1 limit, respectively, in segment s at time t, it must be tamped. If t is the time that a tamping window is occupied, the tamping type is preventive. If it is during the maintenance cycle, the tamping type is corrective. On the other hand, if the value of SL or one of $D^{i},i\in ʆ$ passes the alert limit and planning limit, respectively, but are less than intervention limit and UH1 limit, respectively, the corresponding segment can be considered for preventive tamping based on the OM strategy.

Finally, an optimal tamping scheduling that minimises the total maintenance cost must be developed. Accordingly, the optimal tamping matrix $\kappa^{*}$ and maintenance window vector $\Theta^{*}$ must be determined. This optimisation problem is formulated as a MILP model, which will be covered in detail in the ‘Methodology’ section.

## 3 Methodology

**Fig. 1:**
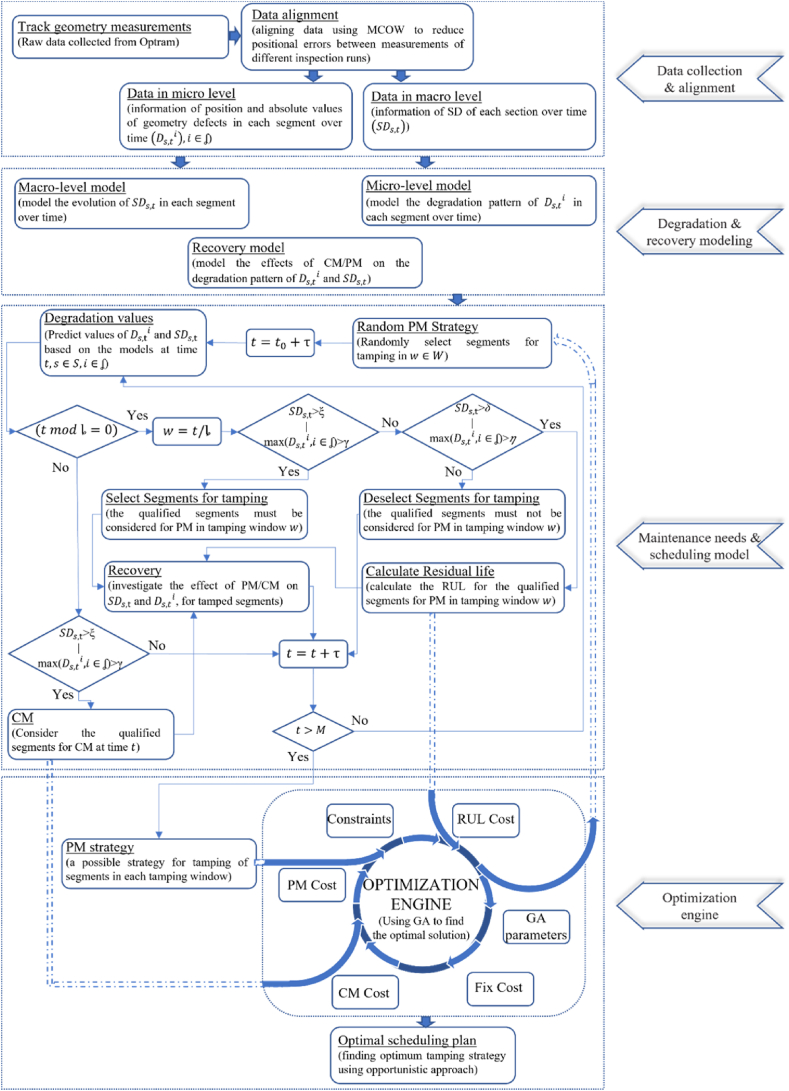
Methodology of the study.

This section presents the methodology for scheduling railway track geometry tamping, as illustrated in Fig. 1. According to this figure, there are four major parts to determining the optimal scheduling plan: data collection and alignment; degradation and recovery modelling; a maintenance needs and scheduling model; the optimisation engine. The details of each component are comprehensively described in Sections 3.1–3.4, with a step-by-step explanation of the inputs, processes, and outputs of the scheduling model.

### 3.1 Data collection and alignment

Track geometry parameters (i.e. longitudinal level, horizontal alignment, gauge, cant, and twist) are widely used to represent track conditions and plan maintenance activities. These parameters are measured by measuring cars at a specific sampling distance (usually 25 cm) whilst running at a certain speed through a track. Generally, geometry measurements suffer from shift, stretching or compression of the measurements, which is called positional error. Such error may arise from either deviation in the position of the inspection data relative to its precise location or a disparity in position between the inspection data and historical data.

**Fig. 2:**
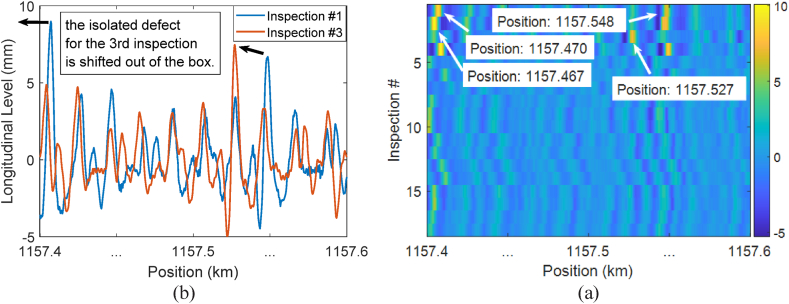
Positional error in track geometry measurements: (a) shift between the measurements of two different inspection runs; (b) shift between the measurements of different inspection runs displayed as a heatmap.

Fig. 2a provides an exemplar of positional error for two inspection runs, wherein the measurements in inspection #3 have undergone a leftward shift, thus making it difficult to trace the changes in single defects over time. Furthermore, the heatmap displayed in Fig. 2b showcases the positional errors present in measurements of track geometry collected during various inspection runs. In this heatmap, colours indicate the magnitude of measurements at their respective positions. Instances of single defects characterised by high positive and negative values are represented by excessively light or dark colours, respectively.

The precise positioning of measurements should ideally result in vertical lines with consistent colours at the same position in the figure. However, Fig. 2b reveals the positional errors between measurements taken during different inspection runs. For instance, a single defect located at position 1157.548 in inspection #1 is found at position 1157.527 in inspection #3, indicating a shift of approximately 21 m between the datasets. The figure further demonstrates that the positions of single defects differ in other inspection runs.

In this study, MFCOW is used for alignment since it showed precise results when aligning track geometry measurements [36].

**Fig. 3:**
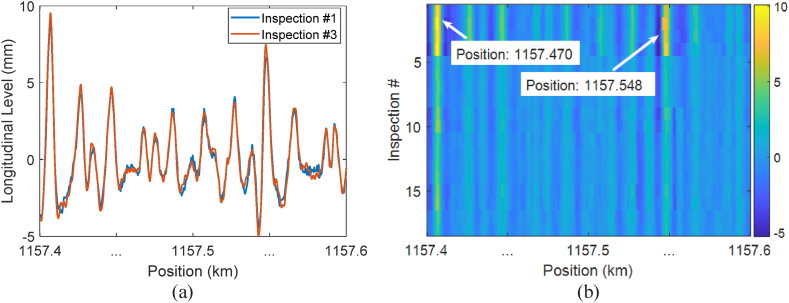
Aligning track geometry measurements: (a) alignment of the datasets presented in Fig. 2a; (b) alignment of the datasets presented in Fig. 2b.

Fig. 3 depicts the alignment of the measurements presented in Fig. 2 using MFCOW. Fig. 3a indicates that MFCOW effectively shifted the measurements in inspection #3 to the right, aligning them with the measurements in inspection #1. Furthermore, Fig. 3b displays vertical lines with consistent colours at the same position, indicating that after alignment, the single defects in each inspection run are located at corresponding positions in other inspection runs. This aligned data enables us to observe the evolution patterns of single defects over time. Readers may refer to the literature [36] for detailed information regarding the MFCOW method and its capabilities.

### 3.2 Degradation and recovery modelling

**Fig. 4:**
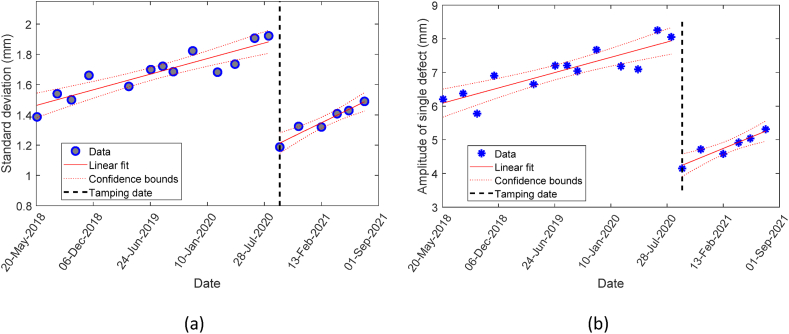
Degradation pattern of standard deviation and a single defect for a sample segment in multiple maintenance cycles.

To monitor the track condition and plan PM actions, not only is the information provided by aggregated quality indicators (e.g. standard deviation) necessary but detailed information regarding the amplitude of single defects, D, over time is also essential. Fig. 4 shows the evolution of standard deviation and a single defect for a sample segment, respectively.

As this figure shows, and by analysing the changes in the value of SL and D in different segments over time, it was observed that both had a linear pattern within a maintenance cycle. It has been proven that despite the simplicity of this model, it has a high performance in modelling track geometry degradation in a maintenance cycle [1]. These observations are in accordance with findings reported by state-of-the-art studies [[37], [38], [39]]. Accordingly, the linear regression models presented in equations (4), (5) are used to model the evolution of SL and D for each segment over time.

(4) $$\begin{array}{cc} {SL}_{s,t}={SL}_{s,0}+b_{s}\left(t-ͳ\right)+\varepsilon_{1} & \forall s\epsilon S \end{array}$$

(5) $$\begin{array}{cc} {D_{s,t}}^{i}={D_{s,0}}^{i}+\beta_{s}\left(t-ͳ\right)+\varepsilon_{2} & \forall s\epsilon S,i\in ʆ \end{array}$$

where ${SL}_{s,t}$ and ${D_{s,t}}^{i}$ denote standard deviation and $i^{th}$ highest peak value of longitudinal level for segment s at time t, respectively. ͳ denotes the time of the latest tamping intervention before time t. ${SL}_{s,0}$ and ${D_{s,0}}^{i}$ are ${SL}_{s,t}$ and ${D_{s,t}}^{i}$ at time ͳ, respectively, whilst $b_{s}$ and $\beta_{s}$ are the degradation rates of SL and D in segment s, respectively. $\varepsilon_{1}$ and $\varepsilon_{2}$ are the Gaussian random error terms with a common mean of 0 and constant variances of ${\sigma_{1}}^{2}$ and ${\sigma_{2}}^{2}$, respectively. That is, $\varepsilon_{1}∼N\left(o,{\sigma_{1}}^{2}\right)$ and $\varepsilon_{2}∼N\left(o,{\sigma_{2}}^{2}\right)$.

To study the evolution of standard deviation and single defects over multiple maintenance cycles, the modelling of tamping recovery is of crucial importance. Performing tamping improves the track geometry condition and decreases the degradation level. Equations (6), (7) show the value changes in standard deviation and single defect after tamping:

(6) $$\begin{array}{cc} {R_{s,t}}^{SL}={{SL}_{s,t}}^{-}-{{SL}_{s,t}}^{+} & \forall s\epsilon S \end{array}$$

(7) $$\begin{array}{cc} {R_{s,t}}^{D^{i}}={D_{s,t}}^{i-}-{D_{s,t}}^{i+} & \forall s\epsilon S,i\in ʆ \end{array}$$

where ${R_{s,t}}^{SL}$ and ${R_{s,t}}^{D^{i}}$ denote recovery values of ${SL}_{s,t}$ and ${D_{s,t}}^{i}$, respectively, ${{SL}_{s,t}}^{-}$ and ${D_{s,t}}^{i-}$ denote the values of ${SL}_{s,t}$ and ${D_{s,t}}^{i}$ immediately before tamping, respectively, and ${{SL}_{s,t}}^{+}$ and ${D_{s,t}}^{i+}$ denote the values of ${SL}_{s,t}$ and ${D_{s,t}}^{i}$ immediately after tamping, respectively. By analysing $R^{SL}$ after numerous tamping actions, it was observed that the value of ${SL}^{-}$ had a strong influence on the tamping quality. In other words, it was observed that a higher ${SL}^{-}$ value can result in a higher $R^{SL}$ value. These observations were in accordance with findings reported by state-of-the-art studies [2,40]. Our observations also showed that the types of tamping, PM and CM, as well as the value of the highest peak (or single defect) in the segment, can greatly affect the recovery quality—especially when performing CM tamping, which covers only a small part of the section affected by a single defect. Considering the influence of tamping type and the value of SL and D before tamping on the recovery of SL, equation (8) was applied to estimate the tamping recovery of SL.

(8) $$\begin{array}{cc} {R_{s,t}}^{SL}=a_{0}+a_{1}{{SL}_{s,t}}^{-}+a_{2}{D_{s,t}}^{i-}+a_{3}\psi +\varepsilon_{3} & \forall s\epsilon S \end{array}$$

Equation (8) includes three explanatory variables: ${{SL}_{s,t}}^{-}$, ${D_{s,t}}^{i-}$, ψ. Notably, ψ is a dummy variable that denotes types of tamping. ${D_{s,t}}^{i-}$ is the highest peak value in the segment before tamping. Additionally, $a_{0}$ is the regression coefficient, whilst $a_{1}$, $a_{2}$ and $a_{3}$ are coefficients for the explanatory variables. $\varepsilon_{3}$ is the Gaussian random error terms with a common mean of 0 and constant variances of ${\sigma_{3}}^{2}$ $\left(\varepsilon_{3}∼N\left(o,{\sigma_{3}}^{2}\right)\right)$.

Since the values of single defects were also predicted in this study, modelling $R^{D^{i}}$ was of utmost importance. By analysing the recovery quality of $R^{D^{i}}$ after both PM and CM in numerous cases, it was observed that the types of tamping, PM and CM, as well as $D^{i-}$ can appropriately represent the recovery quality of $D^{i}$. Accordingly, equation (9) was applied to estimate $R^{D^{i}}$.

(9) $$\begin{array}{cc} {R_{s,t}}^{D^{i}}=\alpha_{0}+\alpha_{1}{D_{s,t}}^{i-}+\alpha_{2}\psi +\varepsilon_{4} & \forall s\epsilon S,i\in ʆ \end{array}$$

where $\alpha_{0}$ is the regression coefficient, and $\alpha_{1}$ and $\alpha_{2}$ are coefficients for the explanatory variables ${D_{s,t}}^{i-}$ and ψ, respectively. $\varepsilon_{4}$ is the Gaussian random error terms with a common mean of 0 and constant variances of ${\sigma_{4}}^{2}$ $\left(\varepsilon_{4}∼N\left(o,{\sigma_{4}}^{2}\right)\right)$.

### 3.3 Maintenance needs and scheduling model

The scheduling model is implemented to optimise the total cost of tamping considering all the maintenance needs. For this purpose, it is essential to define the objective function and its corresponding constraints.

#### 3.3.1 Objective function

Four different costs affect the total cost of tamping: the cost related to the tamping of each segment; the cost of the unused life of segments tamped early; the fixed cost related to each occupied tamping window; the cost of CM. The following objective function (equation (10)) is based on these costs.

(10) $$\min\;E\left(C_{\kappa ,\Theta }\right)=\sum_{w\in W}\sum_{s\in S}\kappa_{s,w}.L_{s}.C^{PM}+\sum_{w\in W}\sum_{s\in S}{Ц}_{s,w}.\kappa_{s,w}.C^{ϻ}+\sum_{w\in W}\Theta_{w}.f+\sum_{m\in M}\sum_{s\in S}\sum_{i\in ʆ}{℘}_{i,s,m}.C^{UH1}$$

The cost of tamping for each segment is related to the length of that segment. The most critical step to calculate the cost of tamping for all the tamped segments in time horizon M is finding the optimal tamping matrix $\kappa^{*}$.The value of $\kappa^{*}$ is determined from a set of possible solutions for κ using an optimisation engine. The optimisation engine will also be used for initialising the matrix κ while ensuring the diversity of solutions of κ. The application of the optimisation engine is explained in detail in Section 3.4. The possible solutions for κ are determined with respect to a set of constraints. These constraints (outlined in the next subsection) are derived from considerations related to track quality, tamping regulations and resource limitations.

#### 3.3.2 Track geometry degradation constraint

Equations (4), (5) are used to find the values of SL and $D^{i}\;\left(i\in ʆ\right)$, respectively, for each segment at the time of each tamping window. Considering the values of SL and $D^{i}$ whilst using the following constraint, matrix κ can be changed based on maintenance needs.

(11) $$\begin{array}{c} \kappa_{s,w}=\left\{ \begin{array}{cc} \begin{array}{c} 1\\ unchanged\\ 0 \end{array} & \begin{array}{c} if\;\left({SL}_{s,t_{w}}>\xi \;|\;\max\left({D_{s,t_{w}}}^{i},i\in ʆ\right)>\gamma \right)\\ if\;\left({SL}_{s,t_{w}}>\delta \;|\;\max\left({D_{s,t_{w}}}^{i},i\in ʆ\right)>\eta \right)\;\&\;\left({SL}_{s,t_{w}}<\xi \;\&\;\max\left({D_{s,t_{w}}}^{i},i\in ʆ\right)<\gamma \right)\;\\ otherwise \end{array} \end{array}\right.\\ \forall w\epsilon W,s\epsilon S \end{array}$$

where $t_{w}$ denotes the time that the tamping window w occurs. Equation (11) expresses that even if the $\kappa_{s,w}\;\left(s\in S,w\in W\right)$ is not considered for tamping in the initial state, it should be planned for tamping because ${SL}_{s,t_{w}}$ passed the intervention limit or at least one of ${D_{s,t_{w}}}^{i},i\in ʆ$ passed the UH1 limit. Additionally, considering this constraint, the initial state should be preserved for $\kappa_{s,w}\;\left(s\in S,w\in W\right)$ if the previous condition is not met and ${SL}_{s,t_{w}}$ passed the alert limit or at least one of ${D_{s,t_{w}}}^{i},i\in ʆ$ passed the planning limit. Finally, there is no need to tamp a segment that has an SL below the alert limit and none of its peaks' values passing the planning limit.

#### 3.3.3 Transition curve constraint

According to the International Union of Railways (UIC) [41], as a practical issue, tamping activities should not be started or finished on curves—especially transition curves. Therefore, it is important to tamp the segments before and after the segments placed on curves that require tamping. Accordingly, matrix κ will be changed considering the following equation:

(12) $$\begin{array}{c} \begin{array}{cc} \kappa_{s-1,w}=\kappa_{s-1,w}\;|\;\left(\kappa_{s,w}\;\&\;\vartheta_{s}\right) & \forall w\epsilon W,s\epsilon S \end{array}\\ \begin{array}{cc} \kappa_{s+1,w}=\kappa_{s+1,w}\;|\;\left(\kappa_{s,w}\;\&\;\vartheta_{s}\right) & \forall w\epsilon W,s\epsilon S \end{array} \end{array}$$

where ϑ is a binary variable that is 1 if track section s is on a curve, and 0 otherwise. Equation (12) ensures that the adjacent segments immediately before and after the segment s, which is on curve, is tamped if s requires tamping.

#### 3.3.4 Middle segment constraint

As another practical issue, to preserve the evenness of the track, it is important to tamp a segment located between two segments that require tamping. Considering this constraint, matrix κ will be changed as shown in the following equation:

(13) $$\begin{array}{cc} \kappa_{s,w}=\kappa_{s,w}\;|\;\left(\kappa_{s-1,w}\;\&\;\kappa_{s+1,w}\right) & \forall w\epsilon W,s\epsilon S \end{array}$$

#### 3.3.5 Tamping time constraints

In each maintenance window, the total tamping time ($T_{w}^{PM})$ must not violate the fixed possession time. The total tamping time includes segments’ tamping times ($T_{w}^{\Omega }$), travelling time ($T_{w}^{\upsilon }$), and the warm-up and cool-down times ($T_{w}^{\omega })$. $T_{w}^{\Omega }$ is calculated based on the number of segments that require tamping ($\kappa_{s,w}=1)$, their lengths ($L_{s})$ and the speed of the machine when tamping ($V_{\Omega })$. $T_{w}^{\upsilon }$ is calculated based on the distance that must be travelled by the tamping machine without tamping the segments with speed ($V_{\upsilon })$. $T_{w}^{\omega }$ is calculated based on the number of cool-down and warm-up events $\phi_{s,w}$ and the corresponding time of each of them $T_{\omega }$. Equations (14), (15), (16), (17), (18) provide the formulas required to ensure that the aforementioned constraints are respected.

(14) $$\begin{array}{cc} T_{w}^{PM}\leq \rho & \forall w\epsilon W \end{array}$$

(15) $$\begin{array}{cc} T_{w}^{PM}=T_{w}^{\Omega }+T_{w}^{\upsilon }+T_{w}^{\omega } & \forall w\epsilon W \end{array}$$

(16) $$\begin{array}{cc} T_{w}^{\Omega }=\sum_{s=1}^{S}\frac{L_{s}}{V_{\Omega }}.\kappa_{s,w} & \forall w\epsilon W \end{array}$$

(17) $$\begin{array}{cc} T_{w}^{\upsilon }=\frac{\sum_{s=1}^{S}L_{s}\left(1-\kappa_{s,w}\right)}{V_{\upsilon }} & \forall w\epsilon W \end{array}$$

(18) $$\begin{array}{cc} T_{w}^{\omega }=\sum_{s=1}^{S}\phi_{s,w}.T_{\omega } & \forall w\epsilon W \end{array}$$

Equation (19) is used to calculate $\phi_{s,w}$.

(19) $$\begin{array}{cc} \phi_{s,w}=\left\{ \begin{array}{cc} \begin{array}{c} \begin{array}{cc} 1 & \left(\kappa_{s,w}-\kappa_{s+1,w}\right)>0 \end{array}\\ \begin{array}{cc} 0 & \left(\kappa_{s,w}-\kappa_{s+1,w}\right)\leq 0 \end{array} \end{array} & s=1,2,\ldots ,S-1\\ \kappa_{s,w} & s=S \end{array}\right. & \forall w\epsilon W \end{array}$$

Considering the above formula, $\phi_{s,w}$ is 1 when $(\kappa_{s,w}-\kappa_{s+1,w}$) is greater than zero and it is zero if $(\kappa_{s,w}-\kappa_{s+1,w}$) is less than or equal to zero for sections 1 to S−1. In addition, for the last section (s=S), the $\phi_{s,w}$ is equal to $\kappa_{s,w}$.

#### 3.3.6 Track geometry restoration constraint

As shown in Fig. 1, considering the newly created κ, the recovery values of tamped segments should be calculated to find the new values of their SL and $D^{i}$ after tamping. Equations (8), (9) are used to calculate the recovery values of SL and $D^{i}$ of tamped segments, respectively. Finally, ${SL}_{s,0}$ and ${D_{s,0}}^{i}$ can be calculated for the tamped segments using equation (20).

(20) $$\begin{array}{c} \begin{array}{cc} {SL}_{s,0}={SL}_{s,t_{w}}-{R_{s,t_{w}}}^{SL} & \forall w\epsilon W,s\epsilon S \end{array}\\ \begin{array}{cc} {D_{s,0}}^{i}={D_{s,t_{w}}}^{i}-{R_{s,t_{w}}}^{D^{i}} & \forall w\epsilon W,s\epsilon S \end{array} \end{array}$$

#### 3.3.7 Unused life constraint

Considering equation (11), some segments may be considered for tamping even though they still did not pass the intervention thresholds. Such early tamping results in unused life for the segments and affects the total cost of tamping. The unused life of the segments in each tamping window $\left({Ц}_{s,t_{w}}\right)$ can be calculated using following equation:

(21) $$\begin{array}{cc} {Ц}_{s,t_{w}}=\frac{1}{b_{s}}\;\left(\xi -{SL}_{s,t_{w}}\right) & \forall w\epsilon W,s\epsilon S \end{array}$$

#### 3.3.8 Fixed cost constraint

Considering Fig. 1 and equation (10), the fixed cost of tamping also has a major effect on the total tamping cost. To consider this cost, if at least one segment is considered for tamping in a tamping window, the corresponding row in vector Θ has a value of 1.

(22) $$\begin{array}{cc} \Theta_{w}=\sum_{s=1}^{S}\kappa_{s,w} & \forall w\epsilon W \end{array}$$

#### 3.3.9 Corrective tamping constraint

However, decreasing the unused life of a segment can decrease the cost of PM, which increases the CM cost. CM is more expensive than PM and may not be as effective as PM. Therefore, to calculate the total tamping cost, finding the potential CMs and their impact on the values of SL and $D^{i}$ whilst also considering the scheduled κ is of utmost importance. Accordingly, as Fig. 1 illustrates, the values of $D^{i}$ for each segment must be estimated and tracked during maintenance cycles. If the values of $D^{i}$ exceed the thresholds γ, CM must be performed and the recovery obtained by CM must be calculated. Since CM affects both the SL of the corresponding segment and the tamped D, the values of all SL and $D^{i}$ before CM must be predicted. For this purpose, considering that track geometry is inspected approximately every 1 month, the values of SL and $D^{i}$ are predicted for each 1 month after tamping window. Equations (4), (5) are used to find the values of SL and $D^{i}\;\left(i\in ʆ\right)$, respectively, for each segment over time. Then, matrix ℘ can be formed using following constraint.

(23) $$\begin{array}{cc} {℘}_{i,s,m}=\left\{ \begin{array}{cc} 1 & if\;\left({D_{s,t_{m}}}^{i}\right)>\gamma )\\ 0 & otherwise \end{array}\right. & \forall m\in M,s\in S,i\in ʆ \end{array}$$

where cells in this matrix, ${℘}_{i,s,m}$ (sϵS, mϵM,i∈ʆ) are binary decision variables and have been defined as 1 if $i^{th}$ defect value of longitudinal level for segment s at time $t_{m}$ is tamped, and 0 otherwise. Considering the created matrix ℘ in each month, equations (8), (9) are used to calculate the recovery values of SL and $D^{i}$ of tamped segments, respectively. This will lead to reset the values of ${SL}_{s,0}$ and ${D_{s,0}}^{i}$ for the corresponding segments using equation (24).

(24) $$\begin{array}{c} \begin{array}{cc} {SL}_{s,0}={SL}_{s,t_{m}}-{R_{s,t_{m}}}^{SL} & \forall m\epsilon M,s\epsilon S \end{array}\\ \begin{array}{cc} {D_{s,0}}^{i}={D_{s,t_{m}}}^{i}-{R_{s,t_{m}}}^{D^{i}} & \forall m\epsilon M,s\epsilon S,i\in ʆ \end{array} \end{array}$$

#### 3.3.10 Binary variables constraints

Finally, the following constraints ensure that $\Theta_{w}$, $\vartheta_{s}$, $\kappa_{s,w}$, $\phi_{s,w}$, ${℘}_{i,s,m}$ are binary variables.

(25) $$\begin{array}{c} \begin{array}{cc} \Theta_{w}\epsilon \left\{0,1\right\} & \forall \;w\epsilon W \end{array}\\ \begin{array}{cc} \vartheta_{s}\epsilon \left\{0,1\right\} & \forall \;s\epsilon S \end{array}\\ \begin{array}{c} \begin{array}{cc} \kappa_{w,s},\phi_{w,s}\epsilon \left\{0,1\right\} & \forall \;w\epsilon W,s\epsilon S \end{array}\\ \begin{array}{cc} {℘}_{i,s,m}\epsilon \left\{0,1\right\} & \forall \;m\epsilon M,s\epsilon S,i\in ʆ \end{array} \end{array} \end{array}$$

### 3.4 Optimisation engine

**Fig. 5:**
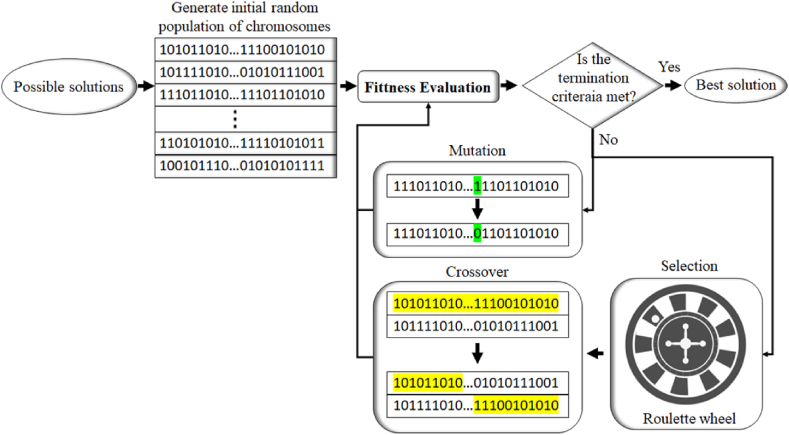
Structure of a genetic algorithm.

Preventive tamping scheduling involves many issues such as safety, cost, serviceability, capacity and passenger comfort, which make it a non-deterministic polynomial-time hard (NP-hard) problem [17,42]. Although there is no suitable provable algorithm for solving NP-hard problems, metaheuristics algorithms such as GA have wide applications in solving these types of problems [2,34]. The GA is recognised as a high-performance solver for NP-hard problems in different fields—including scheduling problems—and is widely used in benchmarking other algorithms [2,17,42,43]. The GA is a practical optimisation tool for scheduling track geometry tamping due to its inherent features and characteristics, such as the ability to find (at least) near-optimal solutions and avoid exhaustive searching [2,42]. Therefore, in this paper, the GA is used as the optimisation engine to find the optimal tamping matrix $\kappa^{*}$. To produce high-quality solutions, the GA relies on the evolutionary generational cycle. To address complex optimisation issues, the GA involves six key phases: initialisation; fitness evaluation; selection; crossover; mutation; termination. In light of these phases, the structure of the GA is illustrated in Fig. 5.

In Fig. 5, chromosomes refer to the segments in each tamping window. Although initialisation can be done randomly, using GA for initialisation ensures the diversity of the initial solutions within a set of possible solutions. To create initial solutions for the current problem, the number of tamping windows that should be occupied and the number of segments that should be tamped in each occupied window are selected randomly.

A random number between 5 and 15 % of the total number of segments determines the number of segments that can be tamped in an occupied window. To convert this initial solution into a feasible solution, the procedure explained in Section 3.3 should be followed. This phase must be iterated until the number of the created solutions equals the required population.

In the next phase, the solutions are ranked based on the cost function (the more cost-effective solutions have a greater likelihood of being selected to produce new solutions). Then, in the other three phases (i.e. selection, crossover and mutation), the algorithm attempts to find new solutions while ensuring the diversity of the solutions. This generational process is repeated until a stopping criterion is passed. The criterion can be a solution that satisfies the minimum criteria, reaches a fixed number of solutions, etc.

## 4 Case study

The proposed model was tested through a case study that used data collected from a heavy haul railway line in Sweden that connects the cities of Luleå and Boden. Although this line is utilised by both passenger and freight trains, the majority of the tonnage transported on this line is related to the mining industry and the transportation of iron ore. The train speeds vary from 50 to 60 km/h for loaded iron ore trains, to 60–70 km/h for unloaded trains and 80–135 km/h for passenger trains. For the case study, an 11-km section of line 119, consisting of 60 track segments of varying lengths, was selected. The length of each track segment was determined by infrastructure managers and typically ranged from 100 to 300 m. The case study analysed data from 48 inspection runs conducted between 2014 and 2021.

**Fig. 6:**
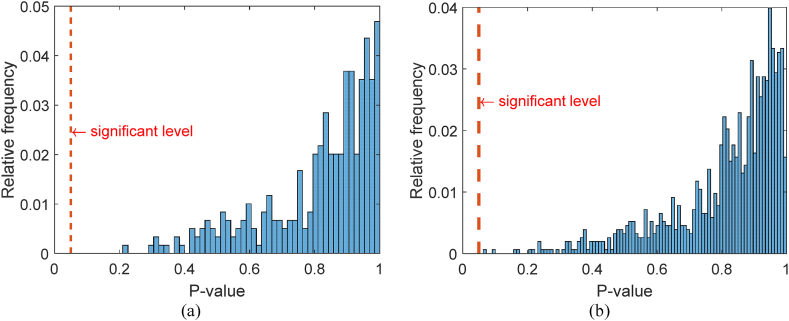
P-value of the KS test for the residuals of the linear model: (a) modelling the evolution of SL; (b) modelling the evolution of D.

The planning horizon was established as 3 years and maintenance windows were scheduled every 6 months. Equation (4) was utilised to predict the value of SL for each track segment in the available maintenance windows. Equation (5) was applied to predict the value of the highest peaks in each segment, which have the potential to become single defects over time. The parameters ${SL}_{s,0},b_{s}$, ${D_{s,0}}^{i}$ and $\beta_{s}$ were taken from the data for each segment. A Kolmogorov–Smirnov (KS) test was conducted to verify the normality assumption of the residuals from the linear models. The results of the test are summarised in Fig. 6a and b, which display histograms of the p-values obtained from Equations (4), (5), respectively. Since the p-value from the KS test is greater than the significance level (0.05), there is no reason to reject the normality assumption of the residuals.

The estimation of the parameters of the recovery models (Equations (8), (9)) was performed using the data obtained from tamping interventions conducted on line section 119. The results of the coefficient estimates are displayed in Table 2, Table 3, respectively. The results of the F-test on the models indicate the significance of the recovery models for SL and D, with p-values of 1.01e-53 and 1.44e-262, respectively. Furthermore, the R-squared values demonstrate that the models can explain approximately 60 and 73 % of the variability in the recovery of SL and D, respectively.

**Table 2:** Estimated parameters for the model in Equation (8).

Parameters		Coefficient	P-value
$a_{0}$		−0.025	0.045
$a_{1}$		0.349	2.873e-14
$a_{2}$		0.021	0.041
$a_{3}$	CM	−0.51	1.5054e-17

**Table 3:** Estimated parameters for the model in Equation (9).

Parameters		Coefficient	P-value
$\alpha_{0}$		−0.68329	2.6012e-30
$\alpha_{1}$		0.663	3.7166e-255
$\alpha_{2}$	CM	−0.23675	0.043089

Furthermore, the initial parameters for developing the model shown in Table 4 were determined through discussions with experts from Trafikverket, and in accordance with the findings of Khajehei, Haddadzade et al. [2]. Additionally, the parameters for the GA were established according to the specifications outlined in Table 5.

**Table 4:** Initial model parameters.

Description	Parameters	Value
Time horizon	M	3 years
Interval of each maintenance cycle	ȴ	6 months
Interval of each inspection	τ	1 month
Fixed possession time for tamping work	ρ	8 h
Warm-up and cool-down times	$T_{\omega }$	20 min
Speed of the tamping machine whilst tamping	$V_{\Omega }$	1 km/h
Speed of the tamping machine during travel	$V_{\upsilon }$	80 km/h
Alert limit for SL	δ	1.2 mm
Intervention limit for SL	ξ	1.5 mm
Planning limit for D	η	8 mm
UH1 limit for D	γ	10 mm
Fixed cost for utilised maintenance window	f	150,000 SEK
Cost of PM (per metre)	$C^{PM}$	25 SEK
Cost of CM (per defect)	$C^{UH1}$	11000 SEK
Cost of the unused life of segments tamped early	$C^{ϻ}$	3000 SEK

**Table 5:** GA parameters.

Parameter	Value
Size of the population	200
Number of generations	300
Crossover percentage	0.9
Mutation percentage	0.5
Mutation probability	0.005

## 5 Results and discussion

**Fig. 7:**
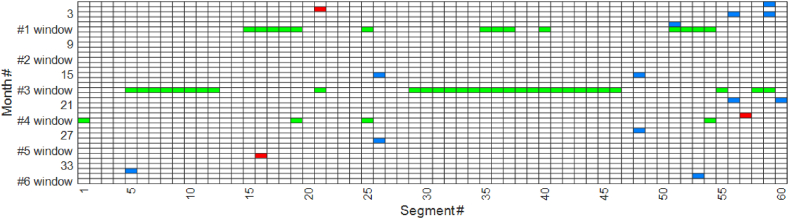
Optimal tamping scheduling (green: PM tamping; blue: CM for only one defect in a segment; red: CM for more than one defect in a segment).

**Fig. 8:**
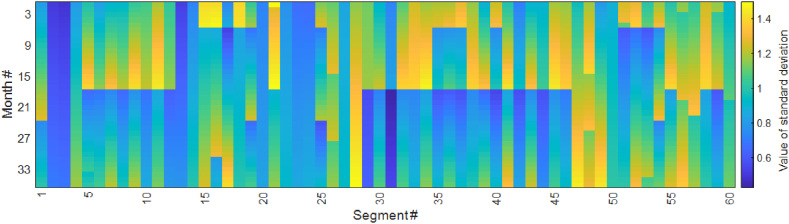
Condition of the segments throughout the entire time horizon.

The optimal tamping schedule was derived using the GA to solve the scheduling problem. The outcomes, which consist of the number of tamping actions for both PM and CM, are presented in Table 6 and illustrated in Fig. 7, Fig. 8. While there were six maintenance windows available, Fig. 7 clearly illustrates that the OM strategy has resulted in the occupation of only three maintenance windows. Moreover, the model made a noticeable effort to group tamping activities as closely as possible, as evidenced by the pattern in the figure.

**Table 6:** Results of the optimal tamping schedule.

Parameter	Value
Number of segments affected by PM tamping (#)	48
Total length affected by PM tamping (metres)	9280
Number of defects affected by CM tamping (#)	25
PM tamping time (hours)	9.2
Travel time (minutes)	42
Warm-up and cool-down times (hours)	4.6
Total tamping time (hours)	14.65
Fixed cost (SEK)	$4.5\times 10^{5}$
Total PM cost (SEK)	$2.32\times 10^{5}$
Total CM cost (SEK)	$2.75\times 10^{5}$
Total unused life cost (SEK)	$1.87\times 10^{5}$
Total cost (SEK)	$11.45\times 10^{5}$

In Fig. 7, we observe that during the periodic inspections (performed every τ months), CM activities were also conducted to address single defects and improve the condition of track segments. This figure indicates that in most cases, only one defect exceeded the threshold γ (as indicated by blue squares), while on three occasions, multiple single defects occurred (as indicated by red squares), resulting in the ${SL}_{s,t}$ threshold (ξ) being exceeded. Fig. 8 illustrates the condition of each segment throughout the entire time horizon based on the ${SL}_{s,t}$ metric, with brighter colours indicating a more severe condition. Examination of this figure reveals that the model successfully prevents the value of ${SL}_{s,t}$ from surpassing ξ, as evident from the absence of bright colours beyond a certain threshold.

An analysis of Table 6 reveals that tamping operations incurred a total cost of approximately 1.145 million SEK, predominantly due to the high fixed cost associated with occupying the tamping window. Interestingly, the cost of CM tamping was found to be higher than that of PM tamping, despite the significantly lower number of CM tamping operations. The data presented in the table suggests that tamping activities were carried out for a total of 14.65 h, with no instances of exceeding the allotted possession time of 8 h per tamping window. Although most of the time was spent on segment tamping, it is worth noting that warm-up and cool-down times also significantly contributed to the overall tamping duration.

**Fig. 9:**
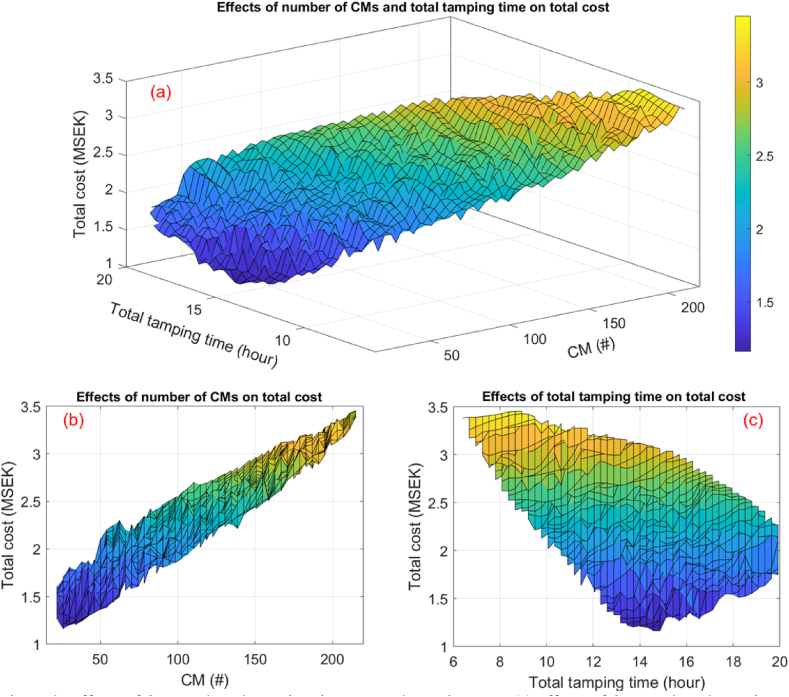
Effects of CMs and total tamping time on total tamping cost: (a) effects of CMs and total tamping time; (b) effects of the number of CMs; (c) effects of total tamping time.

Fig. 9 illustrates the impact of the number of CMs and total tamping time on the total tamping cost. Brighter colours in the figure indicate cases with higher costs. Upon examining Fig. 9a and b, it becomes evident that the number of CMs has a significant influence on the total tamping cost. Hence, finding the optimal number of CMs that minimises the total tamping cost is crucial. For instance, in this case, scheduling tamping with 18 CMs results in the total tamping cost increasing to 1.418 million SEK, representing approximately a 25 % increment.

Additionally, Fig. 9a and c suggest that conducting tamping in less time leads to an increase in the number of CMs and subsequently results in substantially higher tamping costs.

**Fig. 10:**
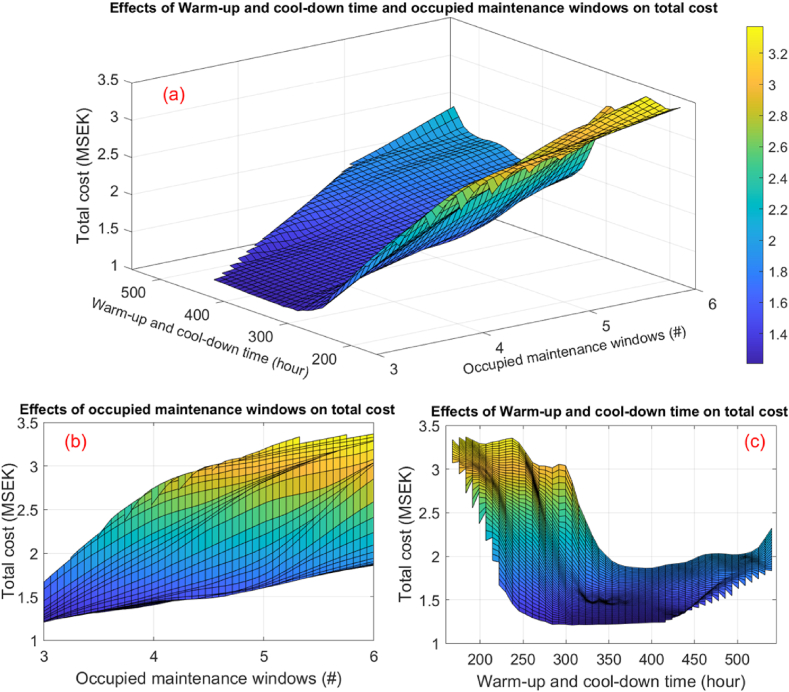
Effects of number of occupied maintenance windows and warm-up and cool-down times on total tamping cost: (a) effects of the number of occupied maintenance windows and warm-up and cool-down times; (b) effects of the number of occupied maintenance windows; (c) effects of warm-up and cool-down times.

Fig. 10 depicts the effects of the number of occupied maintenance windows and warm-up and cool-down time on the total tamping cost. Fig. 10a and b reveal that the number of occupied maintenance windows, which impacts the fixed cost, exerts a substantial influence on the total tamping cost. However, in certain cases, it may be more cost-effective to occupy more maintenance windows to achieve an optimal tamping cost.

Furthermore, Fig. 10a and c demonstrate that excessively low or high warm-up and cool-down times can substantially increase the tamping cost. Consequently, determining optimal warm-up and cool-down times holds immense potential to positively impact the total tamping cost. It is essential to note that the OM strategy's influence on grouping segment tamping directly affects the warm-up and cool-down time. This underscores the crucial role of the OM strategy in attaining an optimal tamping cost.

In conclusion, the study identified fixed cost and the number of CM activities as the most influential parameters in increasing the total tamping cost. Additionally, significant attention to warm-up and cool-down times can considerably reduce the time spent on tamping.

To further comprehend the effects of different parameters on optimal tamping scheduling, an examination of the effects is warranted.

### 5.1 Sensitivity analysis of the effects of maintenance cycle intervals on optimal tamping scheduling

In this section, we investigate the impact of different maintenance cycle intervals on optimal tamping scheduling. The sensitivity analysis evaluates how varying the maintenance cycle interval affects the overall tamping costs and scheduling. Specifically, we examined the effect of three different maintenance cycle intervals (3, 6 and 9 months) whilst keeping all other parameters constant. The data utilised for this sensitivity analysis were sourced from an 11-km section of Line 119 on the heavy haul railway line in Sweden, as described in the case study section.

**Fig. 11:**
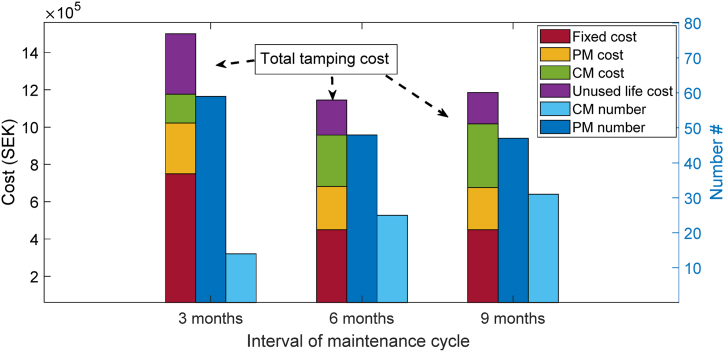
Results of tamping costs and the number of PM and CM activities for scenarios with different maintenance cycle intervals.

Fig. 11 presents the results for tamping costs, including fixed cost, CM cost, PM cost and unused life cost, as well as the number of CM and PM activities obtained for the optimal tamping scheduling when considering different maintenance cycle intervals.

As shown in Fig. 11, a shorter maintenance cycle interval leads to a significant reduction in the number of CM activities but a slightly higher number of PM activities when compared to longer intervals. However, this increased frequency of tamping results in a noticeable increase in total tamping cost due to more occupied tamping windows. On the other hand, when the maintenance cycle interval is too long (9 months), the number of CM activities increases critically, leading to an increase in CM cost and thus the total cost of tamping. As shown in Fig. 11, a 3-month maintenance cycle interval requires 14 CM activities, whereas a 9-month interval requires 31 CM activities, indicating a more than 50 % decrease in the number of required CM activities when using a 3-month maintenance cycle interval.

Notably, 6- and 9-month maintenance cycle intervals resulted in the same fixed cost (450000 SEK for occupying three tamping windows); however, a significant increase in CM cost resulted in a difference in the total cost of tamping. Based on these findings, we conclude that the 6-month maintenance cycle interval strikes a better balance between the number of CM and PM activities and the total cost of tamping.

### 5.2 Sensitivity analysis of the effect of fixed cost on optimal tamping scheduling

The fixed cost of tamping is directly influenced by a company's resources and can vary from contractor to contractor. This cost is generally dependent on factors such as the number of maintenance machines used, fuel consumption, logistics and crew salaries, among other relevant expenditures. To evaluate the impact of different fixed costs on optimal tamping scheduling, we performed a sensitivity analysis. Specifically, we considered three different tamping fixed costs (10000, 50000 and 150000 SEK) whilst keeping all other initial parameters presented in Table 4 constant.

**Fig. 12:**
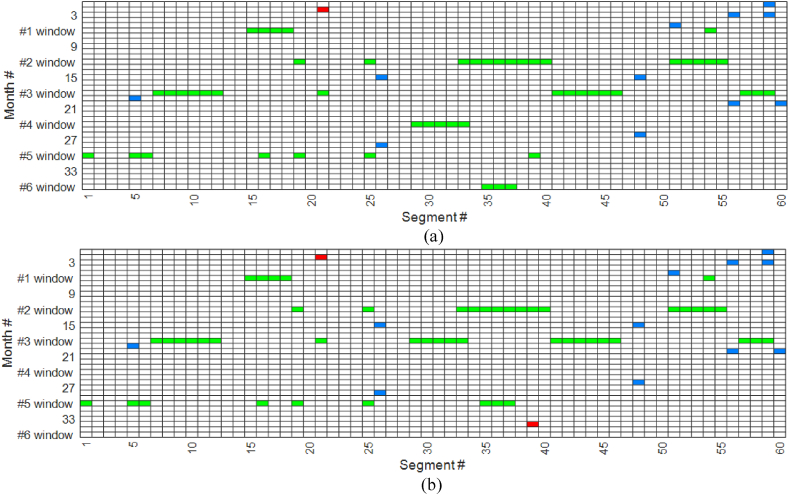
Optimal tamping scheduling with different tamping fixed costs: (a) 10000 SEK; (b) 50000 SEK (green: PM tamping; blue: CM for only one defect; red: CM for more than one defect in a segment).

Fig. 12a and b presents the results for tamping fixed costs of 10000 and 50000 SEK, respectively, and can be compared with the results obtained for a fixed cost of 150000 SEK (see Fig. 7). As demonstrated in these figures, a lower fixed cost results in more occupied maintenance windows. This is because when the fixed cost is low, the impact of occupying a tamping window on the total tamping cost is minimal. Therefore, the model tends to schedule tamping activity for a segment when the value of standard deviation is closer to its upper bound.

The results in Fig. 12a indicate that when the fixed cost is 10000 SEK, all tamping windows are used, compared to Fig. 7, in which only four tamping windows are used. In comparison with Fig. 7, it should be noted that although four tamping windows are used in Fig. 12b, the tamping activities are postponed. This implies that the track segments in Fig. 7 are preserved in better condition over time.

Upon examination of the findings presented in Fig. 7, Fig. 12, it is evident that the number of CM activities varies significantly based on the fixed tamping costs of 10,000, 50,000 and 150,000 SEK. Specifically, the number of CM activities is 16, 19 and 25, respectively, for the corresponding tamping fixed costs. As fixed costs increase, the model tends to shift towards corrective maintenance strategies, which can increase the number of defects before they are addressed but ultimately helps in managing overall costs. These results demonstrate that when the fixed cost for a tamping window is exceptionally high, adopting a preventive tamping strategy for addressing every potential single defect may not be as cost-effective. These findings highlight the importance of considering fixed costs when optimising tamping schedules. In practice, high fixed costs may necessitate fewer but more targeted maintenance actions, while lower fixed costs allow for more frequent preventive actions.

### 5.3 Sensitivity analysis of the effects of unused segment life on optimal tamping scheduling

**Fig. 13:**
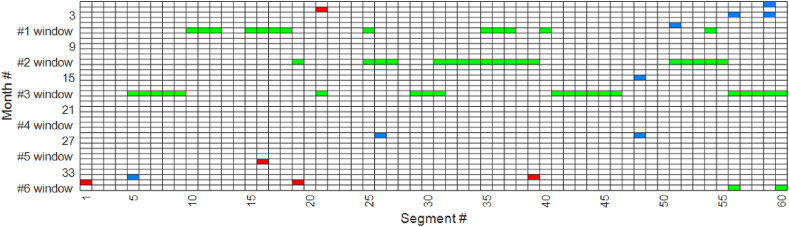
Optimal tamping scheduling without considering the cost of unused segment life (green: PM tamping; blue: CM for only one defect in a segment; red: CM for more than one defect in a segment).

Early tamping performed based on the OM strategy can lead to a reduction in the useful segment life before reaching the upper level for tamping. If early tamping increases the frequency of tamping actions on a segment, it can have a destructive effect on the segment's life cycle. Therefore, the effect of early tamping on the segment life is considered in the model. We analysed the results for optimal tamping scheduling with and without considering the unused segment life. To perform this analysis, we optimised the tamping scheduling whilst excluding the cost of the unused life of segments tamped early in the model (see Fig. 13 and Table 7) and compared it with the model that includes the cost of unused segment life (see Fig. 7 and Table 6). The results reveal that when the cost of unused segment life is not considered in the model, the total tamping cost decreases, as expected, since we excluded a cost parameter from the analysis. However, a notable finding is that the numbers of both PM and CM activities increased. Specifically, there was an approximately 33 % increase in the number of PM activities and a 32 % increase in the number of CM activities. This is because the model tends to perform tamping earlier based on the OM strategy, whilst postponing it may be more cost-effective.

**Table 7:** Results of the optimal tamping schedule.

Parameter	Value
Number of segments affected by PM tamping (#)	53
Total length affected by PM tamping (metres)	12375
Number of defects affected by CM tamping (#)	33
PM tamping time (hours)	9.9
Travelling time (minutes)	41
Warm-up and cool-down times (hours)	5.7
Total tamping time (hours)	16.25
Fixed cost (SEK)	$6\times 10^{5}$
Total PM cost (SEK)	$2.475\times 10^{5}$
Total CM cost (SEK)	$3.63\times 10^{5}$
Total unused life cost (SEK)	0
Total cost (SEK)	$1.21\times 10^{6}$

Based on these insights, it can be concluded that not considering the cost of unused segment life can significantly increase the frequency of both CM and PM activities, consequently leading to an increased possession time for tamping. As such, incorporating the cost of unused segment life in the decision-making process is critical to achieving more efficient tamping scheduling and reducing overall maintenance costs.

## 6 Conclusion

This study developed and optimised an opportunistic tamping schedule to minimise total maintenance costs for railway tracks. For modelling purposes, the standard deviation of the longitudinal level and single defects in each segment were utilised as key track quality indicators. Linear models were employed to represent the evolution of these indicators over time. Additionally, the impacts of both preventive and corrective tamping activities on the values of the indicators were considered in the scheduling model.

The tamping scheduling problem was formulated as a MILP model whilst considering the economic and structural dependence of OM. The objective function of the model included four main components: the tamping cost for each segment; the fixed cost for each occupied tamping window; the cost of the unused life of segments tamped early; the cost of corrective tamping for rectifying single defects. A GA was utilised to find the optimal solution.

The proposed model was evaluated using data from the Main Western Line in Sweden. The findings revealed that the fixed cost of occupying each maintenance window exerted the most significant influence on the total tamping cost. Notably, while most of the possession time for tamping was allocated to the tamping of segments, warm-up and cool-down times also made substantial contributions. Consequently, strategically grouping segments for tamping actions through an OM strategy emerged as essential. Furthermore, the results demonstrated that neglecting the cost of unused segment life in the model led to a considerable increase in the number of both preventive and corrective tamping activities, resulting in an extended possession time for tamping. These findings underscore the importance of incorporating the cost of unused segment life in the decision-making process.

Future studies should explore several potential areas for improvement. Investigating the influence of seasonal changes on the occurrence of single defects within track geometry measurements could reveal valuable insights, particularly how these variations correlate with weather and environmental factors. Expanding the tamping scheduling problem to address network-level concerns presents an intriguing challenge, potentially leading to a more comprehensive approach by incorporating the allocation of maintenance resources within the optimisation framework. Additionally, exploring optimisation strategies for scheduling problems with flexible maintenance cycle intervals could offer substantial potential for refining and enhancing the effectiveness of maintenance scheduling.

## CRediT authorship contribution statement

**Mahdi Khosravi:** Writing – original draft, Visualization, Validation, Software, Methodology, Investigation, Formal analysis. **Alireza Ahmadi:** Writing – review & editing, Supervision, Methodology, Investigation, Funding acquisition. **Ahmad Kasraei:** Writing – review & editing, Methodology. **Arne Nissen:** Resources, Data curation.

## Data availability statement

The data that has been used is confidential.

## Declaration of competing interest

The authors declare that they have no known competing financial interests or personal relationships that could have appeared to influence the work reported in this paper.
